# The Association Between Social Support and Psychological Distress in Latina Mothers Living in Miami-Dade County, Florida

**DOI:** 10.7759/cureus.10848

**Published:** 2020-10-08

**Authors:** Jake W Levine, Pedro Ferrer, Anton J De Witte, Fallon H Levitt, Grettel Castro, Marcia Varella, Patria Rojas, Juan M Acuna

**Affiliations:** 1 Medical and Population Health Sciences Research, Herbert Wertheim College of Medicine, Florida International University, Miami, USA; 2 Biomedical Science, Florida Atlantic University Charles E. Schmidt College of Medicine, Boca Raton, USA; 3 Health Promotion and Disease Prevention and Center for Research on U.S. Latino HIV/AIDS and Drug Abuse, Robert Stempel College of Public Health and Social Work, Florida International University, Miami, USA; 4 Epidemiology and Population Health, Khalifa University, Abu Dhabi, ARE

**Keywords:** social support, psychological distress, acculturation, latina, mental health

## Abstract

Background: This study analysed the relationship between social support and psychological distress in Latina women in Miami-Dade County. Acculturation was examined as a modifying factor.

Methods: A 2005 data set from interviews of 155 Latina mothers in Miami-Dade County, from mother-daughter dyads, was analysed. Social support was measured using the Interpersonal Support Evaluation List (ISEL) score. Psychological distress was based on self-reporting symptoms of depression, anxiety, or suicidality. Acculturation was based on English proficiency and length of U.S. residency.

Results: Compared to those with high social support, women with low social support had greater odds of reporting psychological distress (odds ratio = 7.8 [95% CI 2.70-22.10]). Acculturation did not modify the association (p=0.74).

Conclusions: Social support was inversely associated with psychological distress among Latina women. Acculturation was not an effect modifier, likely due to inadequate power. The study has clinical implications for mental illness prevention in this population.

## Introduction

Anxiety disorders as a whole are some of the most common mental illnesses in the United States, affecting about 18% of adults [1]. Major depressive disorder does not lag far behind, affecting about 6% of adults. Due to the increasing prevalence of these conditions, it is important to identify their mechanisms and risk factors, so that at-risk populations can be targeted with effective interventions.

One of the many risk factors postulated to increase the prevalence of these psychiatric illnesses is low social support [2]. Social support represents the amount of material, psychological, and emotional support that a person receives or perceives being given [3]. This study focuses specifically on perceived interpersonal support, which refers to an individual’s report of interpersonal support available to him or her. This type of support is considered by some to be a more sensitive measure of an individual’s ability to cope with physical and mental health adversity [4].

Studies have shown that a lack of social support may predispose individuals to psychological distress, which is strongly associated with psychiatric illness. This effect is more evident in women, possibly because women may rely more heavily on their friends, relatives, and children for emotional support, while men may draw social support primarily from their spouses [5]. However, the association between social support and psychological distress has not been consistent in all populations, especially immigrants from Latin America [4].

When assessing immigrant populations, the level of acculturation might mediate how psychological distress is affected by various risk factors. Acculturation is the process by which immigrants assimilate into the dominant culture. Latino immigrants are known to maintain stronger and larger social support systems relative to European Americans [5,6]. Therefore, social support might serve as a protective factor for Latino immigrants with low acculturation [4]. Previous studies have attempted to identify this relationship; however, there is significant heterogeneity amongst the literature. Some studies suggest a negative correlation between psychological distress and acculturation; others, a positive one [7]. This is due to the fact that acculturation is a complex process that varies based on an immigrant’s educational level, occupational skill, previous exposure to Western culture, as well as the degree of discrimination in the place one settles in [7].

Social support has been linked to the prevalence of psychological distress in various populations, including Latino immigrants. However, there is a scarcity in the literature showing this link in immigrant Latina mothers within Miami-Dade, Florida, a region known to have considerable cultural heterogeneity [8]. Furthermore, the literature suggests a lack of evidence assessing the specific modifying effect of acculturation on the association between social support and mental health. Overall, this points to the need for further research on the influence of social support and acculturation on psychological distress within this demographic. Thus, our study aims to analyse the association between social support and symptoms of psychological distress specifically in Latina mothers, as well as how acculturation may modify this relationship.

## Materials and methods

Study setting and participants

The study consisted of a cross-sectional, secondary analysis of a database named the Women’s Study (WS) gathered by the Center for Research on U.S. Latino HIV/AIDS and Drug Abuse (CRUSADA) [9]. The WS is a longitudinal study originally aimed at identifying intergenerational transmission of drug use and related health behaviors among Latina mother-daughter dyads residing in Miami-Dade County, Florida [9]. The WS consisted of 316 Latina women, aged 18-78, living in Miami-Dade County, whose mother or daughter wished to participate in a two-hour face-to-face interview. Mother-daughter dyads were then categorized into groups according to the presence of substance abuse. Trained bilingual interviewers conducted interviews, mostly at the participant’s homes. Interviews for the WS were conducted in 2005 and from 2007 to 2010. The responses of the 2005 baseline survey, which had the highest number of participants, were used. In addition, only data of the mothers' were included in this study to eliminate the correlation between responses within the mother-daughter dyad. The final analytical sample included 155 participants. The Florida International University Institutional Review Board approved the study.

Variables

Social support was measured using a truncated version of the Interpersonal Support Evaluation List (ISEL). The original ISEL tool is a 40-item survey that quantifies social support using four categories: tangible, belonging, self-esteem, and appraisal [4]. Each of the four categories have 10 items, totaling 40 items. However, the WS data set quantified social support using only the tangible and belonging subscales, truncating the tool to a 20-item survey. The tangible items subscale score is intended to measure perceived availability of material aid, while the belonging subscale score is meant to measure the perceived availability of people one can interact with [10]. Answers to individual ISEL questions ranged from definitely true (3), probably true (2), probably false (1), to definitely false (0). The scores of the two subcategories are combined to produce a cumulative ISEL score. Based on the tertiles of the scores obtained (ranged 0-80), the ISEL score is categorized into low, medium, and high social support. Low social support corresponds to ISEL scores below the 33rd percentile, medium as scores between the 33rd and 66th percentile, and high scores being above the 66th percentile.

The dependent variable of this study was the occurrence of psychological distress in the past 30 days and lifetime. Psychological distress was considered present if participants reported having either symptoms of depression, anxiety, or suicidal ideation. In the parent study, participants were asked whether they had a period of time (that was not a direct result of alcohol/drug use) in which they had either of the following: (1) experienced serious depression-sadness, hopelessness, loss of interest, or difficulty with daily function; (2) experienced serious anxiety/ tension, feeling uptight, feeling reasonably worried, or experienced inability to feel relaxed; (3) experienced serious thoughts of suicide (participant seriously considered a plan for taking his/her life); and (4) attempted suicide (including actual suicidal gestures or attempts). Responses for these four questions were recorded as “yes” or “no”. Psychological distress was considered present if the participants responded “yes” to any of the four previously stated questions. The absence of the outcome was considered only for those who answer “no” to all four questions. Some of the variables analysed for potential confounding include the following: income, current employment, marriage, health insurance, and age.

Acculturation was assessed for its potential role as an effect modifier. It was defined according to information collected on specific proxy measures: English proficiency composite score, time reported living in the United States, and language of preference used for the WS interview [11]. English proficiency was measured by the participant’s self-recognition of their aptitude in performing the following three tasks in English: writing, speaking, and reading. The scale used ranged as 1 = not at all, 2 = fair, 3 = good, and 4 = excellent. The sum of the answers used for these three tasks equaled a composite score ranging from 3 to 12. Individuals were classified as “more acculturated” if they fulfilled any of the following criteria: English used as the interview language; achieved a score of 12 on English proficiency; lived in the United States for at least 25 years. If individuals did not meet any of the aforementioned criteria, they were classified as “less acculturated.”

Statistical analysis

Exploratory and descriptive data analysis was performed, followed by bivariate analyses to assess for potential confounders in the study. Chi-square testing and analysis of variance (ANOVA) (for categorical and continuous variables, respectively) were performed to assess statistically significant differences in the characteristics according to exposure, and subsequently, to the outcome. Confounders were considered for variables for which a different distribution was found according to exposure and outcome-reflected in a p-value <0.2. This larger alpha was chosen to ensure accounting for as many potential confounders as possible. Multivariate logistic regression analysis was performed to assess for the independent association between levels of social support and psychological distress. Lastly, effect modification by acculturation was tested by the addition of an interaction term (social support*acculturation) in the regression models. Different models were assessed as to report the most parsimonious model with best fit of the data. Significance was considered for p-values <0.05 for a two-tailed test.

## Results

Social support

Social support scores for this sample ranged from 0 to 80. Based on the aforementioned tertiles, social support scores were categorized as low (<62), medium (62-72), and high (>73) social support levels. Age and income were found to be different across the low, medium, and high social support groups. Women reporting medium social support levels were on average five years older than women reporting low and high levels of social support. A larger proportion of women (32%) with low social support reported lower annual income ($0-$4999) compared to women with medium (16.9%) and high (20%) social support. Conversely, a larger proportion of women (44.4%) reporting high social support also reported higher income ($20,000) compared to women with medium (30.5%) and low (12.0%) social support. Lastly, about 24% of women were found to have higher acculturation levels, but no statistically significant differences were found for the frequency of acculturation across the three strata of social support (Table 1).

**Table 1:** Overall sample characteristics according to social support levels *p<0.2. ^a^Analysis of variance performed for difference of means. ^b^Fisher's exact test used because >20% of cases had an expected count <5. ^c^Alcohol consumption includes beer, wine, or liquor.

Characteristics		Total, N (%)	Social support, N (%)			p-value
			Low	Medium	High	
N (%)		155	51 (32.9)	59 (38.1)	45 (29.0)	
Mean age (SD)			49.9 (10.2)	55.0 (10.0)	50.3 (8.6)	0.009*^,^^a^
Income	$0-$4999	35 (22.5)	15 (32.0)	10 (16.9)	9 (20.0)	0.047*
	$5000-$9999	31 (20.0)	12 (24.0)	14 (23.7)	5 (11.1)	
	$10000-$14999	30 (19.4)	10 (20.0)	13 (22.0)	7 (15.6)	
	$15000-$19999	14 (9.0)	6 (12.0)	4 (6.8)	4 (8.9)	
	>$20000	44 (28.4)	6 (12.0)	18 (30.5)	20 (44.4)	
Education	Less than high school	49 (31.6)	20 (39.2)	14 (23.7)	15 (33.3)	0.726
	High school or equivalent	37 (23.9)	11 (21.6)	16 (27.1)	10 (22.2)	
	Some education after high school	41 (26.5)	13 (25.5)	16 (27.1)	12 (26.7)	
	Bachelor's or above	28 (18.1)	7 (13.7)	13 (22.0)	8 (17.8)	
Unemployed		85 (54.8)	34 (66.7)	29 (49.2)	22 (48.9)	0.117*
Latin origin	Caribbean	53 (34.2)	16 (31.4)	20 (33.9)	17 (37.8)	0.214
	Central American	30 (19.4)	12 (23.5)	10 (16.9)	8 (17.8)	
	South American	46 (29.7)	10 (19.6)	23 (39.0)	13 (28.9)	
	Other Latina	26 (16.8)	13 (25.5)	6 (10.2)	7 (15.6)	
Positive criminal history		21 (13.5)	10 (19.6)	5 (8.5)	6 (13.3)	0.235
Not married/no partner		108 (69.7)	39 (76.5)	43 (72.9)	26 (57.8)	0.11*
Had chronic medical problems		72 (46.5)	24 (47.1)	28 (47.5)	20 (44.4)	0.949
Lack health insurance		65 (41.9)	27 (52.9)	25 (42.4)	13 (28.9)	0.058*
No alcohol consumption in the past 3 months^c^		57 (36.8)	22 (43.1)	20 (33.9)	15 (33.3)	0.515
Reported drug abuse		11 (7.1)	4 (7.8)	4 (6.8)	3 (6.7)	0.820^b^
Reported physician visit in the last 12 months		125 (80.6)	41 (80.4)	48 (81.4)	36 (80.0)	0.984
Higher level of acculturation		37 (23.9)	13 (35.1)	13 (35.1)	11 (29.7)	0.91

Psychological distress

Overall, the prevalence of any self-reported psychological distress among the participants of this study was 49% (Table 2). Women with low levels of social support reported psychological distress more frequently (72.5%) than those with medium (45.8%) and high (33.3%) social support scores (p<0.001, for difference of at least one group). In addition to this result, older age, criminal history, and alcohol consumption in the past three months were significantly associated with a higher prevalence of psychological distress.

**Table 2:** Baseline women’s characteristics and frequency of psychological distress *p<0.2

Characteristics		Total, N (%)	Psychological distress, N (%)		p-value
			No	Yes	
N (%)		155	79 (50.1)	76 (49.0)	
Mean age (SD)			49.5 (9.0)	55.5 (9.7)	<0.001*
Social support score	Low	51 (32.9)	14 (27.5)	37 (72.5)	<0.001*
	Medium	59 (38.1)	32 (54.2)	27 (45.8)	
	High	45 (29.0)	30 (66.7)	15 (33.3)	
Income	$0-$4999	35 (22.6)	19 (54.3)	16 (45.7)	0.13
	$5000-$9999	31 (20.0)	17 (54.8)	14 (45.2)	
	$10000-$14999	30 (19.4)	10 (43.3)	20 (66.7)	
	$15000-$19999	14 (9.0)	4 (28.6)	10 (71.4)	
	>$20000	44 (28.4)	25 (56.8)	19 (43.2)	
Education	Less than high school	49 (31.6)	25 (51)	24 (49)	0.95
	High school or equivalent	37 (23.9)	17 (45.9)	20 (54.1)	
	Some education after high school	41 (26.5)	21 (51.2)	20 (48.8)	
	Bachelor's or above	28 (18.1)	13 (46.4)	15 (53.6)	
Employment	Unemployed	85 (54.8)	37 (43.5)	48 (56.5)	0.13*
	Employed	70 (45.2)	39 (55.7)	31 (44.3)	
Latino origin	Caribbean	53 (34.2)	26 (49.1)	27 (50.9)	0.55
	Central American	30 (19.4)	12 (40.0)	18 (60.0)	
	South American	46 (29.7)	26 (56.5)	20 (43.5)	
	Other Latina	26 (16.8)	12 (46.2)	14 (53.8)	
Criminal history	No	57 (37.8)	34 (59.6)	23 (40.4)	0.04*
	Yes	98 (63.2)	42 (42.9)	56 (57.1)	
Marital status	Not married/no partner	108 (70.0)	52 (48.1)	56 (51.9)	0.74
	Married/partner	47 (30.3)	24 (51.1)	23 (48.9)	
Chronic medical problems	No	83 (53.5)	40 (48.2)	43 (51.8)	0.82
	Yes	72 (46.5)	36 (50.0)	36 (50.0)	
Have health insurance	No	65 (41.9)	35 (53.8)	30 (46.2)	0.31
	Yes	90 (58.1)	41 (45.6)	49 (54.4)	
Alcohol consumed in past 3 months	No	57 (36.8)	34 (59.6)	23 (40.4)	0.04*
	Yes	98 (63.2)	42 (42.9)	56 (57.1)	
Drug abuse	No	144 (92.9)	72 (50.0)	72 (50.0)	0.383
	Yes	11 (7.2)	4 (36.4)	7 (63.6)	
Physician visit in the last 12 months	No	30 (19.4)	16 (53.3)	14 (46.7)	0.6
	Yes	125 (80.1)	60 (48.0)	65 (52.0)	
Acculturation level	Less acculturated	118 (76.1)	62 (52.5)	56 (47.5)	0.12*
	More acculturated	37 (23.9)	14 (37.8)	23 (62.2)	

Multivariate analysis

A statistically significant association was found between social support and psychological distress. After adjusting for age, income, and employment, the odds of having psychological distress were 7.8 times higher in those with low social support compared to those with high social support (odds ratio [OR] = 7.79, 95% CI 2.70-22.10). The odds of psychological distress were almost three times higher among participants with medium social support compared to participants with high social support (OR = 2.9, 95% CI 1.2-7.3). Incidentally, younger age (OR = 0.92, 95% CI 0.88-0.96) and lacking employment (OR = 2.57, 95% CI 1.05-6.30) increased the odds of having symptoms of psychological distress compared to older age and employment, respectively (Table 3).

**Table 3:** Unadjusted and adjusted odds ratio for the relationship between social support and psychological distress OR: odds ratio. *Adjusted model includes all variables shown in the table.

		Unadjusted model OR (95% CI)	p-value	Adjusted model* OR (95% CI)	p-value
Social support score	Low	5.29 (2.20-12.66)	<0.001	7.79 (2.70-22.10)	<0.001
	Medium	1.69 (0.76-3.77)	0.202	2.90 (1.15-7.32)	0.02
	High	1.00		1.00	
Age		0.92 (0.89-0.96)	<0.001	0.92 (0.88-0.96)	<0.001
Income	$0-$4999	1.11 (0.45-2.71)	0.822	0.40 (0.12-1.34)	0.14
	$5000-$9999	1.08 (0.43-2.73)	0.865	0.48 (0.14-1.66)	0.24
	$10000-$14999	2.63 (1.00-6.91)	0.05	1.10 (0.35-3.40)	0.87
	$15000-$19999	3.29 (0.89-12.12)	0.073	1.89 (0.42-8.44)	0.41
	>$20000	1.00		1.00	
Employed	No	1.63 (0.86-3.09)	0.13	2.57 (1.05-6.30)	0.04
	Yes	1.00		1.0	

## Discussion

Results from this study suggest an association between social support and psychological distress among Latina immigrant women living in Miami-Dade County, Florida. Also, acculturation did not modify the relationship between social support and psychological distress.

Our analysis indicated that Latina women who had an ISEL score in the lower tertile (<62) had a 7.79 adjusted OR of reporting psychological distress (symptoms of depression, anxiety, and suicidal ideation) when compared to Latina women who scored in the highest tertile of ISEL. This association is strongly supportive of the link between psychological distress and low perceived social support found in recent studies [3,4,12-14]. For example, there appears to be a linear increase in the likelihood of self-reported psychopathology (including major depressive disorder, generalized anxiety disorder, and attempted suicide) with decreasing perceived interpersonal social support using the ISEL tool [3]. Although there is evidence for this relationship, the limited data concerning the Latina community with respect to social support and mental health makes this study of particular use for this special population.

These findings have implications for the development of public health interventions aimed at improving the mental health in this population. Local organizations and government may be inclined to address social support when spearheading campaigns to prevent mental illness in the community. For example, it is well recognized that activities that help build social networks can be a key factor in increasing physical activity and maintaining friendships, both of which can affect mental health [15]. It may be necessary to address social inclusion when designing public spaces, public transportation, housing developments, and other key structures. Previous studies have also demonstrated the importance of social connections for obtaining transportation in Latino immigrants. As an example, carpooling programs have shown promise in facilitating social inclusivity, which may be an option to improve one’s mental well-being [16].

We expected higher levels of acculturation to be associated with low social support and higher psychological distress, since American culture is traditionally thought of as more individualistic compared to the collectivist values seen in Latin countries [17]. In fact, a study reported that individuals becoming more acculturated via spending more than 21 years in the United States had increased odds of expressing psychological distress [17]. However, while our results found higher frequency of psychological distress in those Latinas who were more acculturated compared to the less acculturated (62% vs 47%), the findings were not statistically significant, possibly due to limited power (only 24% of the Latina sample were considered more acculturated). Another possible explanation for the non-significant association between acculturation, social support, and psychological distress could be that only three items were used to measure this variable (interview’s language preference, time in the United States, and English proficiency). Previous studies have shown that acculturation is a multidimensional variable that should preferentially be measured by several constructs such as cultural values and norms [18]. Nevertheless, it could also be due to the unique cultural and ethnic environment of Miami-Dade County where Latinos can communicate effectively in Spanish, are able to work, and acquire social capital without the need to learn English or adapt to the American culture [19]. Therefore, becoming more acculturated might not have as strong an impact as in other geographic locations, which has been mentioned in some of the literature [20].

Limitations in the study were identified. One such limitation was the truncated independent and dependent variables. Originally, the 40-item ISEL questionnaire consisting of four categories. In this study, the questionnaire was truncated to 20 items and two categories. This adjustment to ISEL may affect its generalizability. Another limitation involved the dependent variable of psychological distress. Participants who exhibited symptoms of anxiety, depression, or suicidal ideation were identified in the outcome measure as a single category. Therefore, our study did not analyze the separate associations between these psychological distress factors. It is possible that important differences among these symptoms of psychological distress were not seen, since they were categorized together in the analysis. Additionally, reporting symptoms does not necessarily confirm a diagnosis, so the study speaks more to the level of psychological distress felt by the sample of Latina women, rather than whether or not they have a diagnosed psychiatric illness. One final caveat is that the data set was collected in a different political climate then what exists today. Over the past several years, policies have become less favorable on immigration as a whole [21]. It is unclear how the relationship between social support and psychological distress in this population has evolved in the last decade.

Based on the limitations of the data set described, the authors encourage future studies to examine the evolution of the relationship of social support and psychological distress in this population under the current political climate. Measures should also be taken to include the entire 40-item ISEL scale, as well as outcome data based on clinical measures of psychiatric illness rather than symptoms of psychological distress. Finally, future studies with a larger sample size may have sufficient power to show that acculturation may, in fact, be an effect modifier in this relationship.

## Conclusions

We found evidence for a strong association between social support and psychological distress in Latina women living in Miami-Dade County, Florida. This finding has implications for the development of public health interventions aimed at improving the mental health in this population. Future studies should focus on more robust measures of social support, clinically measurable mental illness, and the effect of changing political climate on the association between social support and psychological distress in this population.
